# Subcellular Accumulation and Depuration of Zinc in Periphytic Algae during Episodic and Continuous Exposures

**DOI:** 10.1007/s00244-022-00971-2

**Published:** 2023-01-06

**Authors:** Pete Cadmus, Ryan J. Friebertshauser, Nayla Rhein, Stephen F. Brinkman, William H. Clements

**Affiliations:** 1 Aquatic Research Section, Colorado Parks and Wildlife, Fort Collins, CO USA; 2 Department of Fish, Wildlife, and Conservation Biology, Colorado State University, Fort Collins, CO USA; 3 Department of Pharmacology and Toxicology, University of Otago, Dunedin, New Zealand

## Abstract

As the severity of extreme precipitation events increases with global climate change, so will episodic pulses of contamination into lotic systems. Periphytic algae represents bioindicator species in most freshwater systems due to their rapid accumulation of toxicants; therefore, it is vital to understand how accumulation in this group differs across temporally variable exposure regimes. The ability to rapidly accrue contaminants has additional implications for the trophic transfer of metals to primary consumers. While dietary toxicity has been studied in algivorous consumers, techniques used to prepare contaminated periphytic algae for consumption have not been compared. This study used a modified subcellular fractionation method to compare the partitioning of zinc (Zn) in periphyton cultures exposed for various durations (cultured in the presence of Zn and 15 min, 24 h, and 48 h exposures). Three exposure groups were additionally depurated over a period of 24 h in order to compare retention of Zn, an important aspect of preparing diets used in dietary toxicity studies. The results not only provide evidence for increased retention by periphytic algae cultured in the presence of Zn but reveal relationships among treatments and subcellular partitioning that suggest time-dependent accumulation and detoxification. These relationships suggest that episodic exposure of periphytic algae to contaminants may pose a greater risk than that of chronic regimes. Based on these results, we additionally advocate for culturing periphytic algae in the presence of contamination to produce a more reliable diet for dietary exposure testing in algivorous organisms.

Due to their rapid growth, sessile nature, and interface with flowing water and sediment (Sabater et al. 2007), periphytic algae is extremely prone to exposure and rapid accumulation of aqueous toxicants and is therefore often used as indicators of water quality and overall health in lotic systems (Fuchs et al. 1997; Holding et al. 2003). Heavy metal accumulation in these organisms has been observed to decrease chlorophyll *a* content, decrease respiration, and alter community diversity (Austin and Deniseger 1985; Crossey and La Point 1988; Nayar et al. 2003). Zinc (Zn), in particular, is a common threat to algae as it is often present in streams surrounding historical mining activity and horticulture (Valencia-Avellan et al. 2017; Conrad et al. 2020) and has been shown to inhibit chlorophyll production as well as growth and cell division (Omar 2002; Yang et al. 2014; Andosch et al. 2015). While a number of studies have revealed physiological and community level effects of heavy metals on periphytic algae, the rate of accumulation, fate of metals within algal cells, and risks to primary consumers are not widely studied. Understanding these aspects across temporal scales is additionally important as different exposure regime (continuous vs episodic) may produce different effects. One method used to further explain these mechanisms and more accurately explain metal accumulation is through the use of subcellular fractionation.

Through differential centrifugation and time-specific tissue digestion and heating, subcellular fractionation can reveal specific partitioning of contaminants within an organism. By quantifying the fractions affected, this method has been used to predict potential toxicity to aquatic organisms (Klerks and Bartholomew 1991; Wallace et al. 2003; Cain et al. 2004) as well as consumers above them within a trophic system (Reinfelder and Fisher 1994; Wallace and Luoma 2003; Rainbow et al. 2007). Evidence of metals in metal-sensitive components such as organelles and cytosolic proteins is thought to induce toxic effects (Wallace et al. 2003), whereas metals bound to metal-binding proteins (i.e., metallothioneins) or concentrated in metal-rich granules infer methods of detoxification and ultimately tolerance (Brown 1982; Klerks and Bartholomew 1991). This may be a function of exposure regime in which continuous exposure during growth leads to a different subcellular profile compared to pulses of toxicants for acute durations. Perhaps most importantly, metal concentrations partitioned in cytosol and proteins are thought to be more bioavailable and thereby present potential accumulation and magnification to consumers compared to non-soluble fractions such as those held in metal concretions (e.g., metal-rich granules) (Reinfelder and Fisher 1994; Wallace and Luoma 2003). Understanding effects of toxicant exposure to organisms as well as their consumers through the lens of subcellular fractionation is particularly informative when investigating effects of dietary exposure of contaminants.

Given the importance of dietary exposure to aquatic insects (Béchard et al. 2008; Xie and Buchwalter 2011; Baudrimont et al. 2017; Hudson et al. 2019) water quality standards risk being under protective if environmentally realistic dietary exposure routes are not included in aquatic toxicology experiments. There is no mandate to include dietary exposure in experiments used for derivation of standards in the USA (United States Environmental Protection Agency 1985). Guidelines for conducting aquatic toxicology experiments, such as those published by the ASTM (American Society for Testing and Materials 1997) and USA Environmental Protection Agency (United States Environmental Protection Agency 2002) could be drastically improved by providing scientists with methods for providing environmentally relevant food sources to algivorous species. The lack of guidance may be, in part, because no standard for preparing contaminated algal diets exists and that a comparison of subcellular partitioning of toxicants in prepared algal tissue has been unavailable. One of the most prevalent methods for contaminating algae is to bathe a culture in a target contaminant for a chosen period of time. This technique has led to successful trophic transfer of toxicants (Irving et al. 2003; Conley et al. 2009; Xie et al. 2010; Xie and Buchwalter 2011; Baudrimont et al. 2017; Hudson et al. 2019) but duration of periphyton bathing varies widely (e.g., 15 min – 9 days) and suggests that the amount and location of toxicant accumulation may vary as well.

Subcellular location and concentration of toxicants likely inform potential loss of function in periphytic algae as well as the bioavailability of toxicants to consumers. This information can also inform the preparation of environmentally relevant diets of periphytic algae in laboratory experiments investigating dietary toxicity. Two factors likely influence concentration and relative abundance of toxicants in algae. First, the duration of exposure can influence whether metals are loosely bound to the exterior of cells or internally accumulated. If used in dietary exposure studies, loose adsorption could lead to loss of toxicant from the periphyton diet into a testing environment thereby reducing the amount of contaminant transferred via dietary means and increasing the amount transferred by aqueous exposure. Second, exposure time may influence which subcellular fractions of the periphyton are accumulating toxicant. If accumulation occurs in non-soluble fractions then the toxicant may be unavailable to consumers. These issues could potentially lead to inaccurate dosing diets, producing poor estimations of concentrations that pose risk to consumers.

This study compared the subcellular partitioning and retention of Zn in periphytic algae across variable exposure regimes. Acute exposures (15 min, 24 h, and 48 h) were conducted to represent episodic, toxicant pulses (e.g., rain events surrounding mine sites, water treatment facility malfunctions, etc.) as well as diet preparation methods most commonly used in dietary exposure studies of algivorous organisms. As an alternative to episodically exposing preparations, periphytic algae was additionally cultured continuously in high levels of Zn to represent continuous exposure during colonization and growth of the algal mat. We hypothesized that algal tissue cultured in the toxicant would retain more contaminant during a period of depuration than would algal tissue acutely exposed. We additionally hypothesized that periphytic algae cultured in the presence of Zn would have a smaller proportion of contaminant bound to the exterior of algal cells compared to the proteins of inner cell walls, cytosolic proteins, and subcellular structures, perhaps best simulating the subcellular profile of a chronically contaminated site.

## Methods

### Periphyton Culture and Exposure

Cobble substrate covered with periphytic algae was collected from a reach of Clear Creek (Jefferson County, Colorado, USA) which has high levels of Zn contamination from historical mining activities. In order to produce non-contaminated cultures used as control and acute exposure groups, substrate was placed in 1 cm of water containing Guillard's growth medium (Guillard 1975) fortified with 1.36 mM silicon using dissolved sodium meta-silicate 9-hydrate in 11.2 cm × 6 cm polyvinyl chloride (PVC) troughs. Solutions were renewed daily. Pumps (Rio 600, Taam Inc., Camarillo, California, USA) provided a recirculating flow of 757 L/hr. Periphytic algae was cultured on 6.25 cm^2^, unglazed porcelain tiles (Cinca Tile Co., Fiães, Portugal). Tiles were arranged close together in culture trays to limit algal growth to the top surface, thereby producing uniform mats. Cultures received 12 h cycles of wide spectrum (Ecolux Plant & Aquarium Wide Spectrum, F40PL/AQ-ECO, General Electric Inc., Boston MA, USA) light. Culture tanks were positioned under two rows of the paired T12 florescent bulbs such that each tile was 120 cm and 150 cm (± 15 cm) from each pair of lights. Zn-cultured periphyton tiles (i.e., chronic exposure group) were prepared as above but with zinc sulfate (ZnSO_4_) added to growth media of Zn-contaminated cultures to a concentration of 1600 µg/L in order to produce concentrations similar to those found in a survey of Colorado mountain streams (Schmidt et al. 2011). Surface area of culture tanks was scrubbed weekly, and a subset (20%) of tiles were replaced with new acid washed tiles to ensure periphytic algae had ample surface area to colonize. All cultures were maintained for three weeks prior to use in any exposure testing or analysis. Throughout the growth period, one water sample was taken from control and Zn treated troughs once every four days to measure aqueous Zn. Periphyton communities were dominated by *Scenedesmus* spp. (personal communication, Sarah Spaulding, United States Geological Survey, Boulder, Colorado, USA).

Immediately following the three-week growth period, a subset of tiles cultured in Zn (*n *= 4) and non-contaminated cultures (*n* = 4) were processed for subcellular fractionation to assess chronic exposure to Zn as well as growth in control conditions. Using a flow-through system, a subset of non-contaminated cultures was then bathed in 1600 µg/L Zn for either 15 min, 24 h, or 48 h to reflect episodic exposure likely to occur downstream of metal-contaminated landscapes as well as approximate common techniques used when preparing contaminated algae used in dietary toxicity trials (Irving et al. 2003; Conley et al. 2009; Xie et al. 2010). The flow-through system consisted of an 850 mL, circular artificial stream (Brinkman and Johnston 2008). Aqueous Zn samples were collected from the artificial stream at 0 min, 15 min, 24 h, and 48 h to ensure stable Zn exposure. Following acute exposure, four tiles from each group were processed for subcellular fractionation. All preparation methods/exposure regimes are listed in Table 1.

**Table 1:** Exposure regimes/preparation methods used

Method	Description
Control	Cultured in non-contaminated Guillard’s growth media
Zn-cultured	Cultured in Guillard’s growth media contaminated with 1600 µg/L
15 min bathed	Cultured in non-contaminated Guillard’s growth media then bathed in 1600 µg/L for 15 min
24 h bathed	Cultured in non-contaminated Guillard’s growth media then bathed in 1600 µg/L for 24 h
48 h bathed	Cultured in non-contaminated Guillard’s growth media then bathed in 1600 µg/L for 48 h

#### Depuration Experiment

In order to assess depuration and retention of Zn, remaining tiles (*n* = 4) from three of the five preparation methods/exposure regimes (Control, Zn-Cultured, and 48 h bathed) were left to depurate for 24 h in an 850 mL, circular artificial stream (Brinkman and Johnston 2008). The artificial stream received non-contaminated, dechlorinated, municipal tap water (Fort Collins, Colorado, USA) from continuous-flow, serial diluters (based on Benoit et al. 1982) delivering 40 mL/min. Following depuration, all tiles were prepared for subcellular fractionation.

### Subcellular Fractionation

To calculate final subcellular Zn concentrations, dry mass of periphyton was first estimated by photoanalysis of live (wet) algal mats. A photograph of each tile was taken using an Olympus Stylus 850 SW (OM Digital Solutions Americas, Inc, Bethlehem, Pennsylvania, USA) under uniform lighting, F-stop, and shutter speed. Photographs were first cropped using ImageJ (v. 1.40 g) (Abramoff et al. 2004) and then analyzed for mean greenness in MATLAB R2009b (MATLAB 2009) by calculating the average green saturation of pixels based on a 255 RGB color model. Dry mass was then estimated using the following equation based on an index of mean greenness:

$${\text{Dry Mass of Algae on Tile }}\left( {\mu {\text{g}}} \right) \, = \, 0.0000{\text{5661 x }}\left[ {{255} - \left( {\text{Mean Greenness}} \right)} \right] \, {-} \, 0.00{33}0{38}0;$$

(*R*^2^ = 0.813, *n *= 13).

**Fig. 1:**
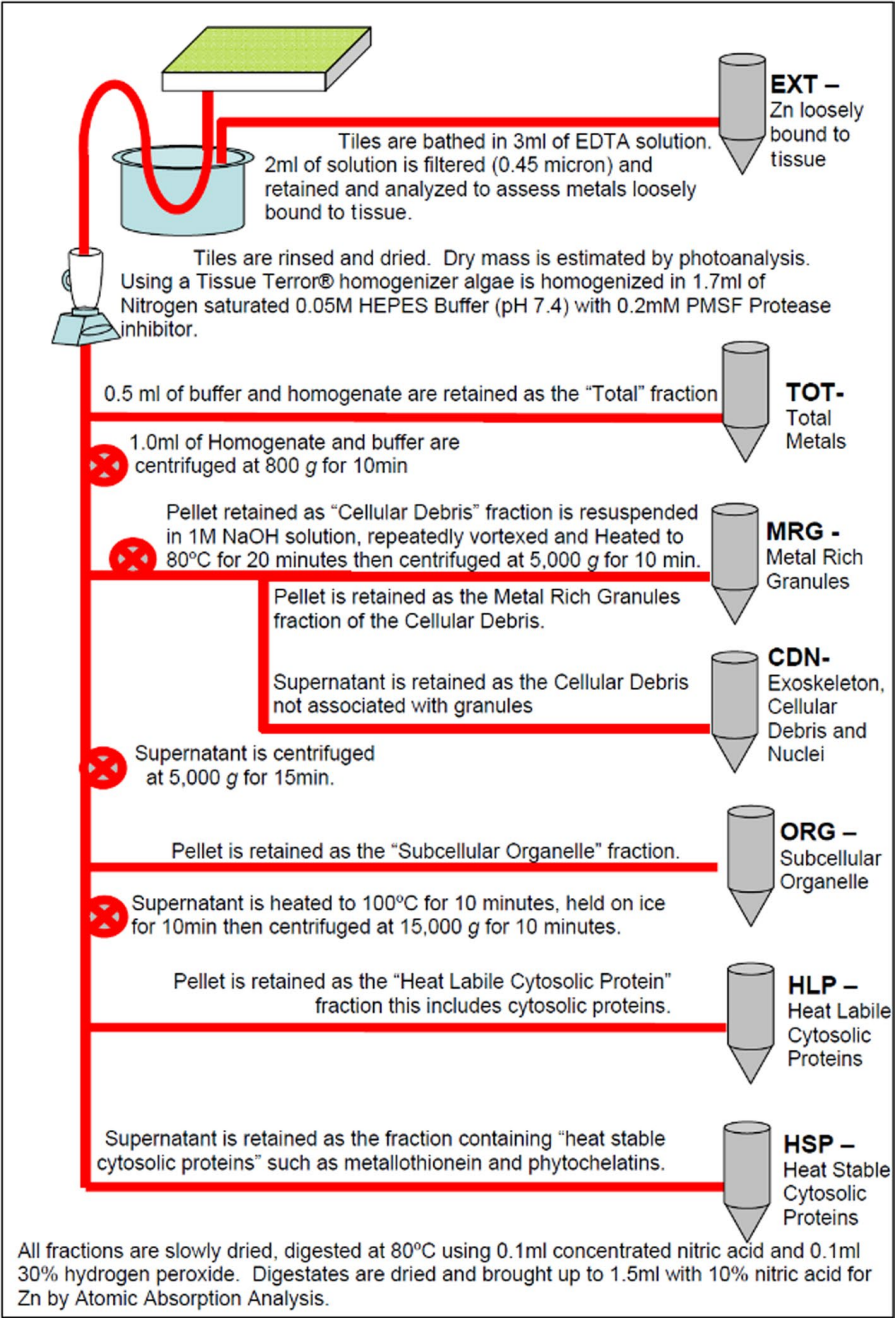
Flowchart describing subcellular partitioning methods

To assess subcellular Zn compartmentalization, periphytic algae from each treatment was partitioned into subcellular fractions through differential centrifugation and heating (Fig. 1). Fractions analyzed included cellular debris and nuclei (CDN) retained in a pellet after 800 × gravity (*g*) (Karp 2010), subcellular organelles (ORG) including mitochondria retained in a pellet after 15,000 g, heat-labile cytosolic proteins (HLP), and heat-stabile cytosolic proteins that include metallothionein (HSP). This method additionally allowed for differentiation between fractions of Zn that were associated with metal-rich granules (MRG) and those loosely bound to the exterior of periphyton and cell fragments (EXT).

Each tile was rinsed with deionized water, air dried on filter paper for 60 s using a Buchner funnel, submerged in 3 ml of 0.01 M magnesium ethylenediamine tetraacetic acid (Mg EDTA) solution, and gently agitated for 60 s to remove loosely bound Zn. Two mL of EDTA solutions were retained in 2-mL polypropylene centrifuge tubes as a measure of Zn loosely sorbed to the surface of tissues (EXT). This fraction was filtered using a 0.45-µm disk filter in order to remove small amounts of algae dislodged during EDTA soaking. While not often considered, metals bound to the EXT fraction are known to cause sublethal effect and are potentially bioavailable to algivorous consumers (Robinson 1998; Taylor et al. 1998; Robinson et al. 2003).

Remaining fractions were partitioned using a modification of methods developed by Bechard et al. (2008), Wallace et al (2003), and Brinkman (2008). Periphyton was homogenized using a Tissue Terror homogenizer (Biospec Products, Inc., Bartlesville, Oklahoma, USA) in 1.7 ml of helium-saturated 0.05 M HEPES Buffer (pH 7.4) with 0.2 mM phenylmethylsulfonyl fluoride protease inhibitor. Homogenate (0.5 mL) was then transferred to a 1.5-mL polypropylene centrifuge tube representing the total metals fraction (TOT) and was only used to calculate percent recovery.

One mL of remaining homogenate was centrifuged at 800 g for 10 min at 4 °C in a 1.5-mL polypropylene centrifuge tube using an Eppendorf 5415 c centrifuge (Eppendorf, Hamburg, Germany). The resulting pellet consisted of cellular debris including tissue fragments, membranes, nuclei (CDN), and metal-rich granules (MRG). This pellet was held at − 20 °C until it was resuspended in 1 M sodium hydroxide (NaOH), repeatedly vortexed while being heated to 80 °C for 20 min, cooled, and then centrifuged at 5000 g for 10 min. The produced supernatant contained resuspended metals not associated with metal-rich granules (CDN) thereby separating it from the pellet containing metal-rich granules (MRG). Each supernatant produced during initial centrifugation was then transferred to a new 1.5-mL polypropylene centrifuge tube and centrifuged at 15,000 g for 10 min at 4 °C. The remaining pellet consisted of subcellular organelles including mitochondria (ORG). The supernatant produced from the second centrifugation was then transferred to a new 1.5-mL polypropylene centrifuge tube, heated to 100 °C for 10 min in order to denature heat-labile proteins. It was then cooled on an ice bath for 10 min and centrifuged at 15,000 g for 10 min. The pellet produced at this step contained heat-labile cytosolic proteins (HLP) while the supernatant represented heat-stabile cytosolic proteins (HSP) such as metallothionein and phytochelating agents (Tripathi and Poluri 2021). Fractions thought to represent metals that are biologically available to consumers consist of ORG, HLP, HSP, and EXT (Taylor et al. 1998; Robinson et al. 2003; Wallace and Luoma 2003).

Throughout subcellular fractionation, all fractions and reagents were chilled on an ice bath to minimize changes in Zn distribution among fractions. Fractions were then dried, digested at 80 °C in 0.1 mL of concentrated nitric acid (Avantor, Radnor, Pennsylvania, USA) for a minimum of 2 h followed by 0.1 mL of 30% hydrogen peroxide and evaporated to dryness. Digestate of subcellular fractions and aqueous samples from testing environments were assessed for Zn using a Video 22 atomic absorption spectrometer (Andover, Massachusetts, USA) with Smith–Hieftje background correction by flame (detection limit of 10 µg/L). Matrix solution for blanks, standards, and dilutions were made of deionized water (Barnstead Nanopure Systems, ThermoFisher, Waltham, Massachusetts, USA). Samples and solutions were preserved with ultra-pure (Ultrex®II, J.T. Baker, Phillipsburg, New Jersey, USA) nitric acid (one drop per 5 ml of sample). Six-point calibration of the atomic absorption spectrometer for each element was conducted prior to each batch of 20 samples and was analyzed after each batch to ensure no drift. Each batch was accompanied with one duplicate sample at the time of collection and one sample split just prior to analysis. Each were flagged if duplicate or split was > 5 or 10% (respectively) from original. Blanks were flagged if greater than 5% of detection limit suggested by manufacturer or detection limit calculated from previous batches. External quality assurance standards for each element were assessed every 10 samples and were flagged if greater than ± 5% from nominal or more frequently at the analyst’s discretion. External standards obtained from nationally certified firms were NIST-Traceable to the SRM 3100 Series. Standards had a certificate of analysis and SDS that guaranteed accuracy (99.999% certified accuracy to ± 0.3%) and stability. If any QAQC flags were observed, the instrument was recalibrated, and all samples of that batch were reanalyzed. Samples found above the highest standard in the calibration curve were diluted (1:2, 1:5 or 1:10) and reanalyzed in a subsequent batch.

### Statistical Analysis

Subcellular proportions of Zn as well as the summed concentration of all fractions were compared across preparation treatments using a one-way analysis of variance. Treatment means were compared using PROC GLM, LSMEANS, and REQWQ in SAS (v. 9.1.3, 2004). Descriptive statistics and 95% confidence intervals were generated using the PROC MEAN procedure. Comparisons of Zn concentration pre- and post-depuration were analyzed in the same manner as above. Type III sum of square was used if replicates were unbalanced. An alpha criterion of 0.05 was used for all analyses. Bar graphs were produced using the ggplot2 package (v. 3.3.5) (Wickham 2016) in R (v. 4.0.3) (R Core Team 2021).

## Results

### Depuration Experiment

**Fig. 2:**
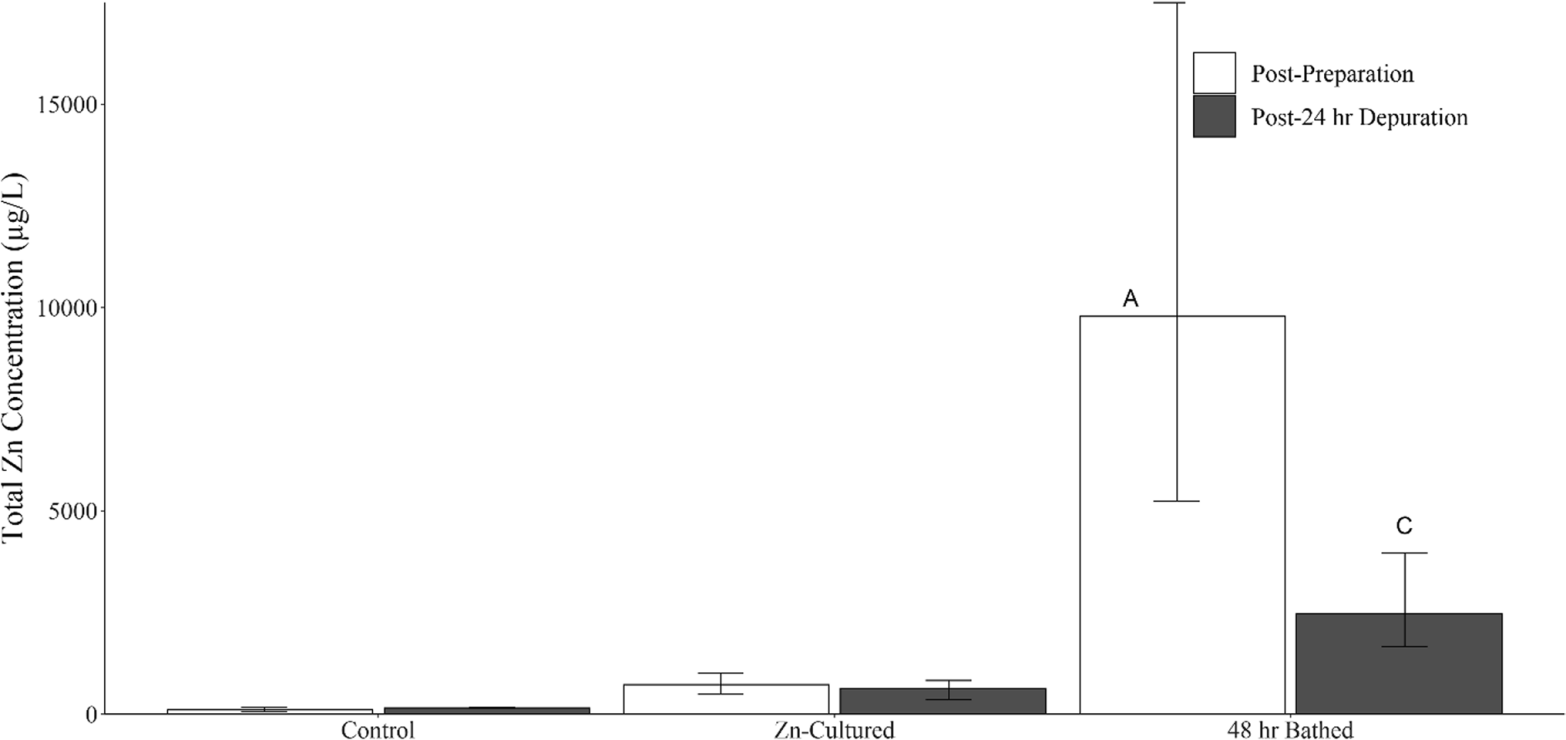
Total Zn concentrations before and after 24 h depuration in in 0 µg/L Zn. Error bars denote standard deviation. Text annotations denote statistical significance (*α* = 0.05)

**Fig. 3:**
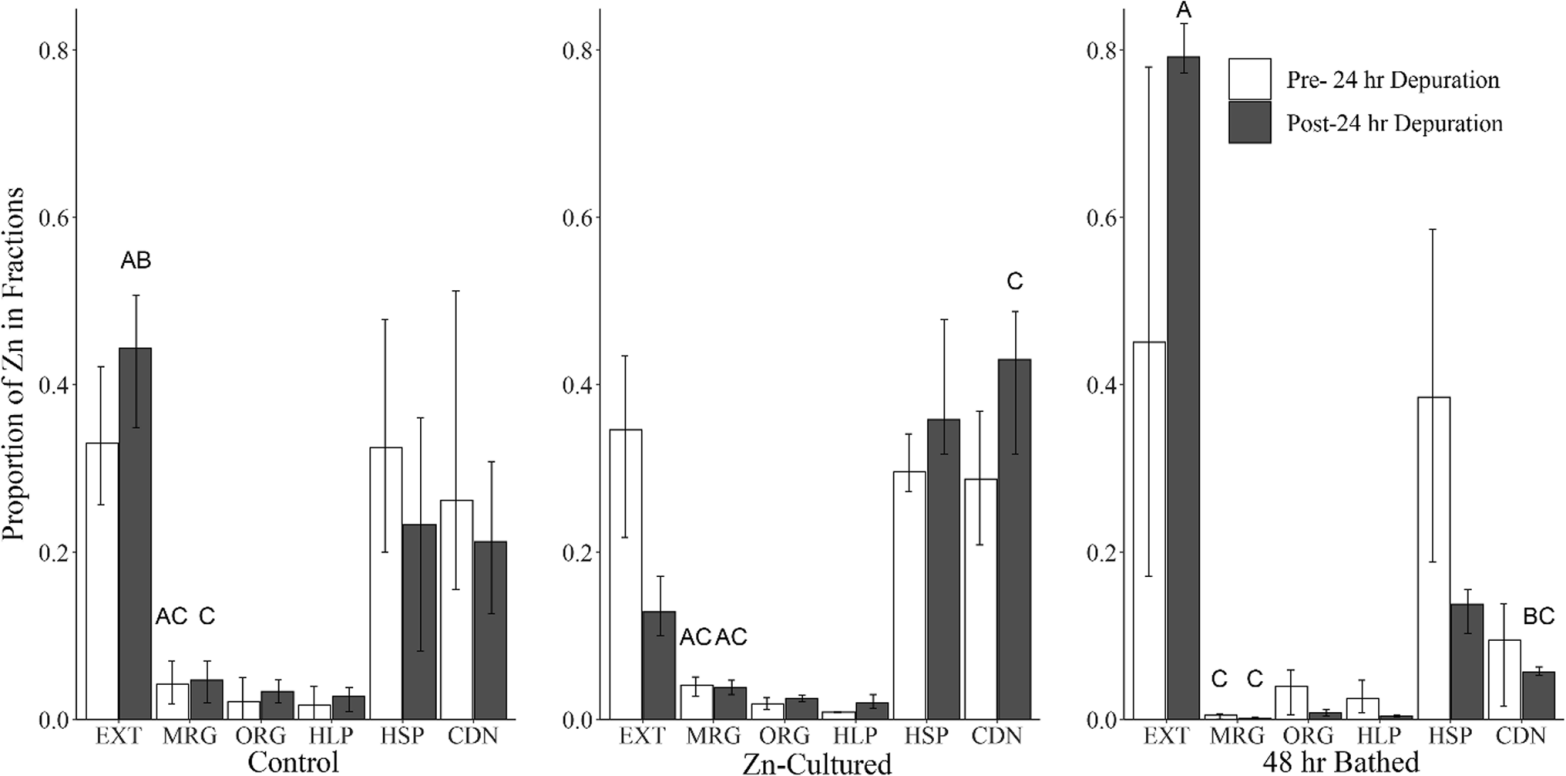
Proportions of Zn in subcellular fractions before and after 24 h depuration in 0 µg/L Zn. Error bars denote standard deviation. Text annotations denote statistical significance (*α* = 0.05)

Periphytic algae cultured in the presence of Zn lost significantly less total Zn post-depuration compared to preparations exposed for 48 h (14% and 75% loss, respectively) thereby supporting our initial hypothesis of improved retention in this treatment (Fig. 2). No statistically significant differences were found when comparing proportions of subcellular fractions before and after 24 h of depuration among any of the three preparation methods tested (Fig. 3). While not statistically significant, it is important to note that algae exposed to Zn for 48 h showed larger portions of Zn in the EXT fraction pre- and post-depuration compared to control and Zn-cultured preparations.

### Subcellular Fractions

When comparing proportions of subcellular Zn in each fraction across preparation methods, few differences were observed (Table 2). Algae exposed for 48 h showed significantly greater Zn concentrations when summing all fractions compared to those cultured in Zn, exposed for 15 min, and the control group. Proportion of Zn in the EXT fraction was significantly higher in tiles exposed for 15 min compared to all other methods. Periphytic algae bathed for 15 min contained significantly higher Zn in the CDN fraction compared to those cultured in Zn. Proportions of Zn in the MRG fractions were significantly higher in Control and Zn-cultured treatments compared to all other methods. No statistically significant differences were found across preparation methods in the ORG, HLP, or HSP fractions. Percent recovery of the subcellular fraction method used was 107.22% (95% CI = 101–113).

**Table 2:** Total Zn concentrations across preparation methods as well as exposure concentrations in preparation troughs

Preparation method	Mean aqueous Zn in preparation troughs (µg/L)	Total concentration (all fractions) (µg/g)	Proportion of Zn in EXT fraction	Proportion of Zn in MRG fraction	Proportion of Zn in ORG fraction	Proportion of Zn in HLP fraction	Proportion of Zn in HSP fraction	Proportion of Zn in CDN fraction
Control	69.3 (23–123)	104.2 (56.3–164) B	0.331 (0.26–0.42) BC	0.043 (0.019–0.07) AB	0.022 (0–0.05) A	0.017 (0–0.04) A	0.325 (0.2–0.478) A	0.262 (0.156–0.512) ABC
Zn-cultured	1672 (1407–2430)	722.6 (502.2–1006) B	0.347 (0.218–0.435) BC	0.041 (0.028–0.051) AB	0.019 (0.012–0.026) A	0.009 (0.008–0.01) A	0.297 (0.273–0.342) A	0.288 (0.209–0.369) AB
15 min bathed	1624 (1583–1647)	815.7 (674.8–1016.6) B	0.758 (0.69–0.844) A	0.005 (0.003–0.008) C	0.017 (0.008–0.025) A	0.009 (0.005–0.012) A	0.178 (0.104–0.272) A	0.033 (0.02–0.057) C
24 h bathed	1624 (1583–1647)	5857.2 (2164.2–13,671.1) AB	0.421 (0.176–0.554) BC	0.005 (0.004–0.008) C	0.028 (0.015–0.064) A	0.027 (0.01–0.075) A	0.379 (0.274–0.476) A	0.14 (0.036–0.273) BC
48 h bathed	1624 (1583–1647)	9780.2 (5238.1–17,494) A	0.451 (0.172–0.78) BC	0.005 (0.001–0.007) C	0.04 (0.006–0.059) A	0.025 (0.008–0.047) A	0.385 (0.189–0.586) A	0.095 (0.016–0.139) BC

Accumulation in fractions is presented as portions of the total concentration. 95% confidence intervals are provided parenthetically for total concentrations and proportions. Range is provided for aqueous Zn concentration in preparation troughs. Capital letters indicate statistical significance (*α* = 0.05)

## Discussion

This study compared the retention and subcellular partitioning of Zn across temporally disparate exposure regimes that additionally represent common techniques used to prepare periphytic algae for use in dietary toxicity testing. Exposure duration, concentration of contaminant, community composition, water quality, and contaminant type differ in nature and will consequently vary accumulation (Vymazal 1984; Howard and Hacker 1990; Lee et al. 1998). Therefore, producing an environmentally relevant algal diet or attempting to simulate site-specific exposure is dependent on the system in question and therefore cannot be standardized. However, examining temporally distinct exposure regimes through the lens of subcellular fractionation offered novel insight into the dynamics of metal accumulation and detoxification mechanisms in these organisms. Given the importance of periphytic algae to algivorous fish and invertebrate species, this insight may allow managers to better predict the risk of dietary exposure to metals after toxicant pulses and researchers to better prepare algal diets for use in dietary toxicity tests.

### Temporal Trends in Accumulation

Our hypothesis that the subcellular profile (locations of accumulation) of periphytic algae cultured in Zn would mimic that of a control condition was not supported by these data. However, certain relationships between treatment and subcellular partitioning (Table 2) suggest time-dependent accumulation and detoxification. The significantly higher proportion of the EXT fraction observed in algae exposed for 15 min demonstrates that acutely exposed algae, as would be seen in an episodic toxicant pulse, simply adsorb contaminant to exterior tissues as opposed to internal accumulation. While not statistically significant, EXT fractions relatively decreased with an increase in exposure duration suggesting time-dependent accumulation. Conversely, the MRG fraction was more highly represented in control and Zn-cultured treatments compared to all others. As this fraction is thought to reflect detoxification of metal (Brown 1982; Klerks and Bartholomew 1991), these results suggest that exposure duration not only effects accumulation but the ability to mount detoxification strategies as well. It is likely that use of MRG to sequester metals in a cell is a longer-term detoxification strategy (> 48 h) than up-regulation of proteins associated with the HSP fraction. A final temporal trend can be seen in the relative increase of HSP fractions with time across acute exposure treatments. The HSP fraction includes metallothionein and phytochelating agents (Tripathi and Poluri 2021) that, like MRG, represent detoxification strategies used in the maintenance of metals homeostasis (Sharma et al. 2016). While the relatively lower HSP proportion in algae cultured in the presence of Zn is counterintuitive to this statement, the relationship between this trend and that of MRG partitions suggests that metallothionein-based detoxification may temporally precede detoxification by metal-rich granules (Table 2).

The relationships stated above suggest that the duration of metals exposure may have a strong bearing on toxic effects to periphytic algae. Metals loosely adsorbed to exterior tissues (EXT) are thought to incur sublethal effects (Robinson 1998; Taylor et al. 1998; Robinson et al. 2003). Inversely, if periphytic algal species are accumulating metals but do not have time to mount a detoxification mechanism than these circumstances may result in more deleterious effects than that of chronic exposure. Although counterintuitive, these data suggest that acute, episodic exposure events may have stronger effect on periphytic algae than sustained chronic exposure. As predictions for climate change suggest the intensification of extreme rainfall events (IPCC 2013), contaminated landscapes will be more prone to episodic transfer of toxicants into adjacent waterways (Batson et al. 1996; Strømseng et al. 2009; Valencia-Avellan et al. 2017; Conrad et al. 2020). This ultimately creates a regulatory concern in that predictable and continuous pollution at contaminated sites may be less of a risk to the periphytic algae than episodic pulses that might go unnoticed by even routine water quality sampling. Further work should investigate subcellular accumulation, fitness impacts, and overall community effects at temporal scales mimicking actual episodic pulses of toxicants seen in nature.

### Trophically Available Metal

While the above relationships may determine the immediate effect to algal communities, they are additionally applicable to the aquatic trophic network. As periphytic algae forms the base of most freshwater trophic systems, understanding their dynamics of metals accumulation has crucial implications to primary consumers and the ecosystem as a whole. Since metals are thought to be more readily assimilated by consumers when bound to certain subcellular portions of their diet (Rainbow et al. 2004; Béchard et al. 2008), subcellular fractionation offers a more refined perspective of trophically transferred contaminants compared to quantifying total metal concentrations alone. Trophically available metal (TAM) (Wallace and Luoma 2003) refers to subcellular partitions containing contaminant that is readily accumulated by consumers. While consumer toxicity may depend on taxa and metal (Wallace and Luoma 2003), certain generalizations surrounding TAM have emerged. For example, fractions such as organelles, enzymes, and metallothioneins have repeatedly shown to be readily absorbed by consumers while cellular debris and metal-rich granules have been observed undigestible (Nott and Nicolaidou 1993; Wallace and Lopez 1997; Wallace and Luoma 2003; Sánchez-Marín and Beiras 2017). The observed difference in bioavailability of metals via metallothioneins and metal-rich granules (Wallace and Lopez 1997) is especially interesting as they are both detoxification pathways (Brown 1982). Trophic transfer of metals is commonly studied through dietary exposure studies involving algivorous macroinvertebrates (Irving et al. 2003; Conley et al. 2009; Xie et al. 2010; Xie and Buchwalter 2011; Baudrimont et al. 2017; Hudson et al. 2019); however, techniques used to prepare algal diets are not standardized or well-studied. When preparing diets for use in dietary exposure tests on algivorous organisms, the concentration of the diet should ideally fluctuate little during the course of the trial, contain biologically available contaminant, and have subcellular distributions similar to that of a non-contaminated, reference culture.

#### Preparation of Periphytic Algae for Dietary Exposure Testing

As discussed above, most fractions thought to represent TAM did not significantly differ across exposure treatment used in this study. However, the EXT fraction of tiles exposed for 15 min were significantly higher than those cultured in the presence of Zn. This discrepancy in partitioning of metals represents the first dosing inaccuracy that may be produced by briefly exposing algal tissues. The second type of dosing inaccuracy was observed through increased depuration over 24 h in tiles bathed for 48 h (Fig. 2). This loss of contaminant into the testing environment has been observed in dietary toxicity studies that prepared periphyton diets by bathing (Conley et al. 2009; Xie et al. 2010). Depuration of metals during testing not only reduces concentrations of dietary exposure but potentially increases accumulation via aqueous exposure thereby obscuring the conclusions that can be made from each exposure route. While the observed depuration in acutely exposed periphytic algae align with loss seen in the literature, the large differences in total Zn concentration between tiles exposed for 48 h and those cultured in the presence of Zn (both pre- and post-depuration) are not easily explained. It is possible that Zn-cultured preparations employed a detoxification mechanism prior to depuration testing that could not be exploited by the acutely exposed group. It is also possible that this discrepancy is an artifact of delivery techniques to each preparation (static renewal in Zn-cultured verses flow-through). While this work describes course differences between different exposure regimes, we suggest that when conducting dietary exposure tests, users should seek to understand the subcellular partitioning and retention of samples collected from environments they wish to simulate.

## Conclusion

Using a modified subcellular fractionation method, this study described subcellular partitioning of Zn in periphytic algae exposed to Zn for variable durations, ranging from acute (15 min) to chronic (cultured in Zn). Acutely exposed groups primarily adsorbed Zn to external tissues while chronically exposed preparations showed larger proportions internally. Accumulation in certain subcellular fractions of chronically exposed preparations suggests that the ability to mount detoxification mechanisms is also dependent on exposure time. Relative to sustained exposure, episodic events may pose a greater risk to periphyton communities if they are unable to mount these defenses.

Observations post-depuration across control tiles, those bathed for 48 h, and preparations cultured in Zn shed light on the retention of contaminant across common strategies used to prepare a periphyton diet for dietary toxicity experiments. Periphyton cultured in Zn showed the highest retention and thereby represents the most reliable method for preparing periphyton used in dietary toxicity testing. While the subcellular fractions thought to represent TAM did not vary across treatments, we believe further investigation into TAM across exposure durations is warranted. While this work describes differences of accumulation and retention across exposure regimes in the laboratory setting, significant work remains to compare laboratory prepared periphytic algae mats to field collected periphyton. This study shows the exposure regime in nature plays a significant role in toxicant accumulation. So too might habitat, taxonomic structure, phenology, and other factors difficult to recreate in the laboratory. We strongly advocate for the use of subcellular fractionation when attempting to understand the impacts of metals to periphytic algae and when attempting to create environmentally relevant food sources for test organisms.

## References

[CR1] Abramoff M, Magelhaes P, Ram S (2004). Image processing with image. J Biophotonics Int.

[CR2] American Society for Testing and Materials (1997) Standard guide for conducting acute toxicity tests on materials with fishes, macroinvertebrates, and amphibians. In: Standard E729 in Vol. 11.05 of the Annual Book of ASTM Standards. American Society for Testing and Materials Agency, West Conshohocken, Pennsylvannia

[CR3] Andosch A, Höftberger M, Lütz C, Lütz-Meindl U (2015). Subcellular sequestration and impact of heavy metals on the ultrastructure and physiology of the multicellular freshwater alga *Desmidium swartzii*. Int J Mol Sci.

[CR4] Austin A, Deniseger J (1985). Periphyton community changes along a heavy metals gradient in a long narrow lake. Environ Exp Bot.

[CR5] Batson VL, Bertsch PM, Herbert BE (1996). Transport of anthropogenic uranium from sediments to surface waters during episodic storm events. J Environ Qual.

[CR6] Baudrimont M, Andrei J, Mornet S (2017). Trophic transfer and effects of gold nanoparticles (AuNPs) in *Gammarus fossarum* from contaminated periphytic biofilm. Environ Sci Pollut Res.

[CR7] Béchard KM, Gillis PL, Wood CM (2008). Trophic transfer of Cd from larval chironomids (*Chironomus riparius*) exposed via sediment or waterborne routes, to zebrafish (*Danio rerio*): Tissue-specific and subcellular comparisons. Aquat Toxicol.

[CR8] Benoit DA, Mattson VR, Olson DL (1982). A continuous-flow mini-diluter system for toxicity testing. Water Res.

[CR9] Brinkman SF (2008). Cadmium toxicity, accumulation, and subcellular distribution in gills and kidneys of brown trout.

[CR10] Brinkman SF, Johnston WD (2008). Acute toxicity of aqueous copper, cadmium, and zinc to the mayfly *Rhithrogena hageni*. Arch Environ Contam Toxicol.

[CR11] Brown BE (1982). The form and function of metal-containing “granules” in invertebrate tissues. Biol Rev.

[CR12] Cain DJ, Luoma SN, Wallace WG (2004). Linking metal bioaccumulation of aquatic insects to their distribution patterns in a mining-impacted river. Environ Toxicol Chem.

[CR13] Conley JM, Funk DH, Buchwalter DB (2009). Selenium bioaccumulation and maternal transfer in the mayfly *Centroptilum triangulifer* in a life-cycle, periphyton-biofilm trophic assay. Environ Sci Technol.

[CR14] Conrad SR, Santos IR, White SA (2020). Elevated dissolved heavy metal discharge following rainfall downstream of intensive horticulture. Appl Geochemistry.

[CR15] Crossey MJ, La Point TW (1988). A comparison of periphyton community structural and functional responses to heavy metals. Hydrobiol.

[CR16] Fuchs S, Haritopoulou T, Schäfer M, Wilhelmi M (1997). Heavy metals in freshwater ecosystems introduced by urban rainwater runoff — monitoring of suspended solids, river sediments and biofilms. Water Sci Technol.

[CR17] Guillard RRL (1975). Culture of phytoplankton for feeding marine invertebrates. Culture of Marine Invertebrate Animals.

[CR18] Holding KL, Gill RA, Carter J (2003). The relationship between epilithic periphyton (biofilm) bound metals and metals bound to sediments in freshwater systems. Environ Geochem Health.

[CR19] Howard CL, Hacker CS (1990). Effects of salinity, temperature, and cadmium on cadmium-binding protein in the grass shrimp, *Palaemonetes pugio*. Arch Environ Contam Toxicol.

[CR20] Hudson ML, Costello DM, Daley JM, Burton GA (2019). Species-specific (*Hyalella azteca* and *Lymnea stagnalis*) dietary accumulation of gold nano-particles associated with periphyton. Bull Environ Contam Toxicol.

[CR21] IPCC (2013) Climate Change 2013: The physical science basis. Contributions of working group I to the fifth assessment report of intergovernmental panel of climate change. Cambridge University Press, Cambridge, United Kingdom and New York, NY, USA

[CR22] Irving EC, Baird DJ, Culp JM (2003). Ecotoxicological responses of the mayfly *Baetis tricaudatus* to dietary and waterborne cadmium: Implications for toxicity testing. Environ Toxicol Chem.

[CR23] Karp G (2010). Cell and molecular biology: concepts and experiments.

[CR24] Klerks PL, Bartholomew PR (1991). Cadmium accumulation and detoxification in a Cd-resistant population of the oligochaete *Limnodrilus hoffmeisteri*. Aquat Toxicol.

[CR25] Lee BG, Wallace WG, Luoma SN (1998). Uptake and loss kinetics of Cd, Cr and Zn in the bivalves *Potamocorbula amurensis* and *Macoma balthica*: effects of size and salinity. Mar Ecol Prog Ser.

[CR26] Nayar S, Goh BPL, Chou LM, Reddy S (2003). In situ microcosms to study the impact of heavy metals resuspended by dredging on periphyton in a tropical estuary. Aquat Toxicol.

[CR27] Nott JA, Nicolaidou A (1993). Bioreduction of zinc and manganese along a molluscan food chain. Biochem Physiol.

[CR28] Omar HH (2002). Bioremoval of zinc ions by *Scenedesmus obliquus* and *Scenedesmus quadricauda* and its effect on growth and metabolism. Int Biodeterior Biodegradation.

[CR29] R Core Team (2021) R: A language and environment for statistical computing.

[CR30] Rainbow PS, Ng T, Shi D, Wang WX (2004). Acute dietary pre-exposure and trace metal bioavailability to the barnacle *Balanus amphitrite*. J Exp Mar Bio Ecol.

[CR31] Rainbow PS, Amiard J-C, Amiard-Triquet C (2007). Trophic transfer of trace metals: subcellular compartmentalization in bivalve prey, assimilation by a gastropod predator and in vitro digestion simulations. Mar Ecol Prog Ser.

[CR32] Reinfelder JR, Fisher NS (1994). The assimilation of elements ingested by marine planktonic bivalve larvae. Limnol Oceanogr.

[CR33] Robinson KA (1998). The influence of prey-surface contamination on aquatic invertebrate predators with contrasting modes of feeding.

[CR34] Robinson KA, Baird DJ, Wrona FJ (2003). Surface metal adsorption on zooplankton carapaces: implications for exposure and effects in consumer organisms. Environ Pollut.

[CR35] Sabater S, Guasch H, Ricart M (2007). Monitoring the effect of chemicals on biological communities. The biofilm as an interface. Anal Bioanal Chem.

[CR36] Sánchez-Marín P, Beiras R (2017). Subcellular distribution and trophic transfer of Pb from bivalves to the common prawn *Palaemon serratus*. Ecotoxicol Environ Saf.

[CR37] Schmidt TS, Clements WH, Cade BS (2011) Identifying limits on stream insect density exposed to metals in the presence of co-limiting factors. North American Benthological Society.

[CR38] Sharma R, Bhardwaj R, Handa N (2016). Responses of phytochelatins and metallothioneins in alleviation of heavy metal stress in plants: An overview. Plant Met Interact Emerg Remediat Tech.

[CR39] Strømseng AE, Ljønes M, Bakka L, Mariussen E (2009). Episodic discharge of lead, copper and antimony from a Norwegian small arm shooting range. J Environ Monit.

[CR40] Taylor G, Baird DJ, Soares AMVM (1998). Surface binding of contaminants by algae: consequences for lethal toxicity and feeding to *Daphnia magna* straus. Environ Toxicol Chem.

[CR41] Tripathi S, Poluri K, Hasanuzzaman M (2021). Phytochelatin-assisted mechanism of heavy metal detoxification in microalgae. Approaches to the Remediation of Inorganic Pollutants.

[CR42] United States Environmental Protection Agency (1985) Guidelines for deriving numerical standards for the protection of aquatic organisms and their uses.

[CR43] United States Environmental Protection Agency (2002), Methods for measuring the acute toxicity of effluents and receiving waters to freshwater and marine organisms (4303T); U.S. Environmental Protection Agency: Washington, DC

[CR44] Valencia-Avellan M, Slack R, Stockdale A (2017). Effect of episodic rainfall on aqueous metal mobility from historical mine sites. Environ Chem.

[CR45] Vymazal J (1984). Short-term uptake of heavy metals by periphyton algae. Hydrobiologia.

[CR46] Wallace WG, Lopez GR (1997). Bioavailability of biologically sequestered cadmium and the implications of metal detoxification. Mar Ecol Prog Ser.

[CR47] Wallace WG, Luoma SN (2003). Subcellular compartmentalization of Cd and Zn in two bivalves. II. Significance of trophically available metal (TAM). Mar Ecol Prog Ser.

[CR48] Wallace WG, Lee BG, Luoma SN (2003). Subcellular compartmentalization of Cd and Zn in two bivalves. I. Significance of metal-sensitive fractions (MSF) and biologically detoxified metal (BDM). Mar Ecol Prog Ser.

[CR49] Wickham H (2016). ggplot2: elegant graphics for data analysis.

[CR50] Xie L, Buchwalter DB (2011). Cadmium exposure route affects antioxidant responses in the mayfly *Centroptilum triangulifer*. Aquat Toxicol.

[CR51] Xie L, Funk DH, Buchwalter DB (2010). Trophic transfer of Cd from natural periphyton to the grazing mayfly *Centroptilum triangulifer* in a life cycle test. Environ Pollut.

[CR52] Yang H, Huang ZY, Li J, Hu Y (2014). MT-like proteins: Potential bio-indicators of *Chlorella vulgaris* for zinc contamination in water environment. Ecol Indic.

